# Effect of intra‐ and inter‐tumoral heterogeneity on molecular characteristics of primary IDH‐wild type glioblastoma revealed by single‐cell analysis

**DOI:** 10.1111/cns.13396

**Published:** 2020-06-02

**Authors:** Zujian Xiong, Qi Yang, Xuejun Li

**Affiliations:** ^1^ Department of Neurosurgery Xiangya Hospital Central South University Changsha China; ^2^ Xiangya School of Medicine Central South University Changsha China; ^3^ Hunan International Scientific and Technological Cooperation Base of Brain Tumor Research Xiangya Hospital Central South University Changsha China

**Keywords:** primary IDH‐wild type glioblastoma, single‐cell analysis, tumoral heterogeneity

## Abstract

**Aims:**

To reveal the effects of intra‐ and inter‐tumoral heterogeneity on characteristics of primary IDH‐wild type glioblastoma cells.

**Methods:**

Single‐cell RNA‐seq data were acquired from the GEO database, and bulk sample transcriptome data were downloaded from the TCGA database with clinical information. Neoplastic subtype and glioma stem‐like cells (GSCs) were identified by matching 5000 random virtual samples based on ssGSEA. CNV was inferred to compare the heterogeneity among patients and subtypes by infercnv. Transition direction was inferred by RNA velocity, and lineage trajectory was inferred by monocle. Regulon network of cells was analyzed by SCENIC, and cell communication was identified by CellPhoneDB.

**Results:**

Glioblastoma (GBM) cells could be divided into four subtypes by Verhaak classifier. However, classification of three subtypes (except NE subtype) was more suitable for GBM cells, and Verhaak classifier has difficulty in distinguishing GSCs. GBM heterogeneity and GBM cells’ regulon network were mainly influenced by inter‐tumoral heterogeneity. Within the same patient, different subclones exist in the same subtype of cells whose transition direction could be predicted by regulon similarity. Apart from inter‐tumoral heterogeneity, different subtype of cells share common subtype‐specific cell‐cell communications.

**Conclusions:**

Inter‐tumoral heterogeneity contributes mainly to GBM heterogeneity and cell molecular characteristics. However, the same subtype of cells shared cell communication similarities.

## 1 INTRODUCTION

Tumor heterogeneity consists of intra‐tumoral and inter‐tumoral heterogeneity, which poses a major challenge in glioblastoma (GBM) diagnosis and treatment.^1^ Robust transcriptome and epigenome studies have revealed the inter‐tumoral heterogeneity of GBM, which is associated with distinct outcomes or therapeutic responses.^2^, ^3^ During tumor progression, neoplastic cells from the same tumor but different locations will acquire different additional mutations or exhibit specific phenotypic or epigenetic states.^1^, ^4^, ^5^, ^6^

Glioblastoma is one of the most fatal and malignant central nervous system (CNS) tumors in adults, with a median overall survival of 15 months.^7^ Multiple observations based on high‐throughput sequencing data revealed tumor heterogeneity of GBM as well as its area‐specific patterns of genomic imbalance, which contributes to prognostic outcome and treatment response.^2^, ^8^ To develop an accurate treatment strategy and improve the therapeutic outcome, many classification methods according to key molecular events and genetic alterations were discovered, among which Verhaak classifier is generally accepted.^9^, ^10^ Although traditional bulk tumor sequencing approaches have identified essential genes and pathways that play important roles in GBM tumorigenesis, they provide limited insights into the cellular diversity and molecular complexity of tumor cells. Recent developments in single‐cell analysis methods and sequencing of individual cells provide a more comprehensive way to explore molecular changes at the cellular level.^11^ Herein, we used scRNA‐seq data of primary IDH‐wild GBM to thoroughly explore the intra‐ and inter‐tumoral heterogeneity by identifying cell subtypes and then compared the difference of transcription factor regulon network and cell communication with the same subtype of cells or immune cells among patients.

## 2 MATERIALS AND METHODS

### 2.1 Datasets and data processing

A total of 3589 cells from four primary IDH wild‐type glioblastoma patients and 430 cells from five primary IDH wild‐type glioblastoma patients’ single‐cell RNA‐seq data according to Smart‐seq2 protocol (GSE84465, GSE57872) were downloaded from the GEO database (https://www.ncbi.nlm.nih.gov/geo).^8^, ^12^ GSE84465 was mainly analyzed, while GSE57872 was used as the validation dataset. A total of 367 samples of TCGA AffyU133a gene expression array data and 143 samples of Illumina HiSeq RNA‐seq data of primary IDH wild‐type glioblastoma and corresponding phenotype data were downloaded from the TCGA database (https://tcga‐data.nci.nih.gov/) via Xena Browser developed by UCSC. The QC of single‐cell RNA‐seq data was performed by scater R package.^13^ Genes expressed in at least two cells were retained. Mitochondrial (MT) genes were set as internal reference. Cells with total counts <25 000 or total genes >6000 and the percentage of MT genes >20 were removed. The scImpute R package was used for imputation, and normalization was conducted by scran R package.^14^ RNA‐Seq data were normalized by transcripts per kilobase million (TPM) method for further analysis.

### 2.2 Subtype and glioma stem‐like cells (GSCs) identification

Subtypes of GBM cells, bulk samples, and GSCs were identified by ssgsea.GBM.classification R package.^15^ First, we generated numerous virtual samples by randomly selecting expression values of the same gene from samples as a virtual sample corresponding gene expression. Then, the ssGSEA scores for each category were calculated. We set 5000 virtual random samples and correlated these samples with the real sample and counted the number of matches with random samples under each subtype. We defined the subtype as the one that had the fewest matches to the random sample. If more than one subtype shared the min matches in one sample, we defined the sample as MIX. We defined the first 10% of min matched neoplastic cells or the sample with match number <3 as mGSCs or pGSCs. The four GBM subtypes (classical, mesenchymal, proneural, and neural) signatures acquired from^10^ and three GBM subtypes (classical, mesenchymal, and proneural) signatures improved by Wang.^15^ Mesenchymal and proneural GSCs (mGSCs and pGSCs) signatures were from.^16^

### 2.3 CNV evaluation and subclone cluster

The CNV evaluation based on single‐cell RNA‐seq raw counts was conducted by infercnv R package. We chose hidden Markov model to predict the CNV states. Gene location data were from AnnoProbe R package. Subclones of specific subtypes were divided by hierarchy clustering based on CNV. Subclone was clustered by SC3 R package.^17^

### 2.4 Differential state potency and cell cycle state prediction

Differential state potency of single‐cell data was predicted by LandSCENT R package. Cell cycle of single‐cell data was predicted by scran R package. The input data were first transferred into SingleCellExperiment class object and normalized by scater R package.

### 2.5 Dimensionality reduction and GO enrichment analysis

A total of 2000 variable genes among all neoplastic cells were found by Seurat v3 R package based on TPM. PCA and Tsne were conducted by Seurat v3 and PCs selected by JackStraw function. Genes with PC value in top 20% were selected for GO analysis by clusterProfiler R package.^18^

### 2.6 Pseudo‐time lineage trajectory and R velocity

RNA velocities were computed via velocyto.^19^ Glioblastoma cells of different subclones and subtypes were used for velocyto analysis to evaluate the state transformation direction. Lineage trajectory plot based on variant feature identified by Seurat v3 was generated by monocle R package.^20^

### 2.7 Regulon and cell communication network identification

To further analyze transcription factor regulons, we adopted SCENIC R package,^21^ using default parameters. For visualization, we mapped the regulon activity (AUC) scores to the TSNE plot and heat map. Intra‐tumoral cell‐cell communication network based on potential receptor‐ligand interaction was inferred by CellPhoneDB from single‐cell transcriptomic data.^22^

### 2.8 Survival and statistical analyses

**FIGURE 1:**
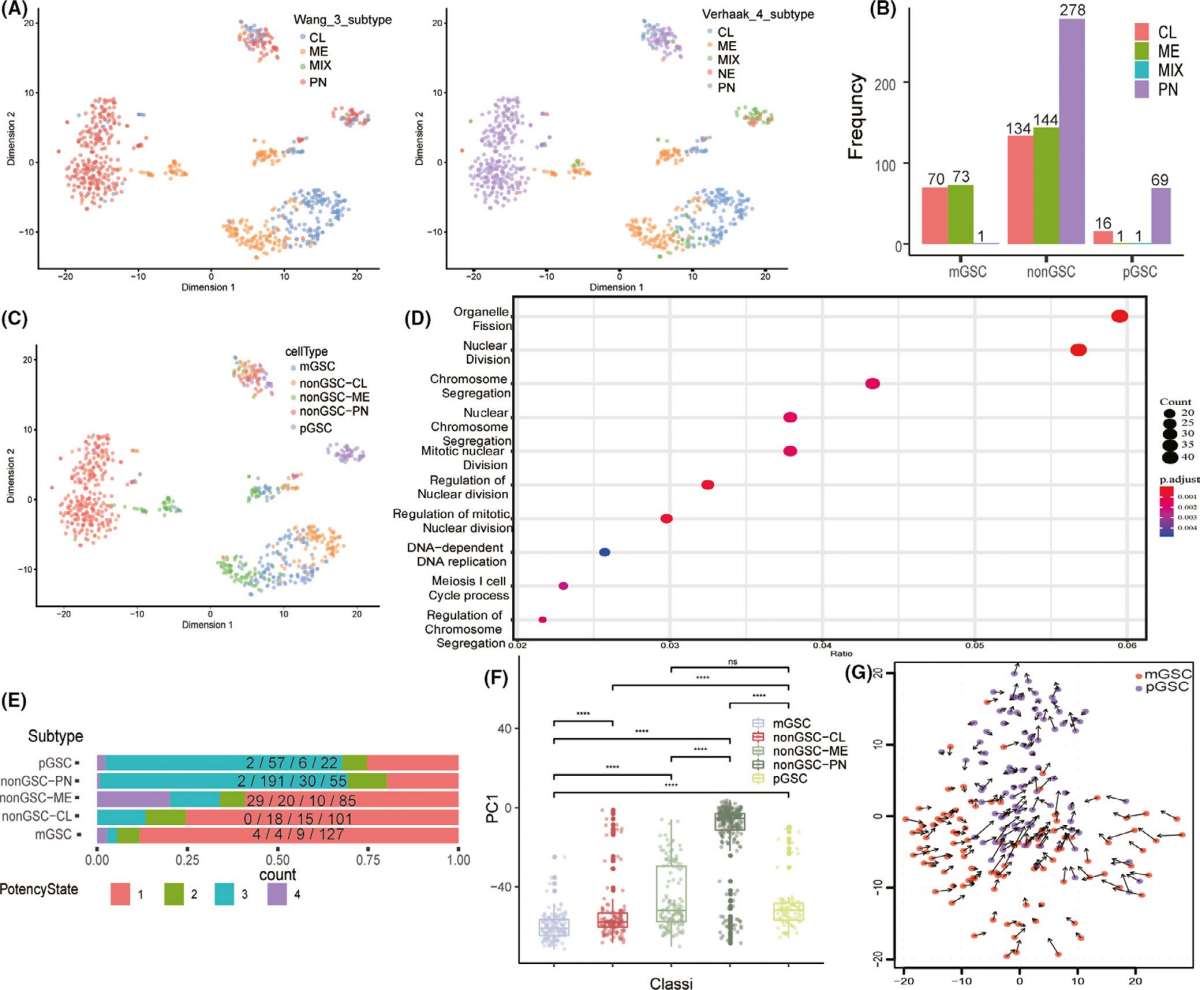
A, the tsne plot on GSE84465 single‐cell dataset revealed the subtypes of each neoplastic cells. CL, classical; ME, mesenchymal; PN, proneural; NE, neural; MIX: cells divided into at least two subtypes. B, distribution of GSC and non‐GSC subtypes. GSCs, glioma stem‐like cells. C, cell subtype classification and distribution. mGSC, mesenchymal GSC; pGSC, proneural GSC. D, GO enrichment pathway based on genes with top 400 PC1 values among all neoplastic cells. E, proportion of cells with different differentiation potency in each subtype, potency state 1 means the cell with the highest differentiation potency, while state 4 means the lowest differentiation potency. F, distribution of PC1 sum in each subtype. G, transition direction between mGSC and pGSC inferred by RNA velocity

**FIGURE 2:**
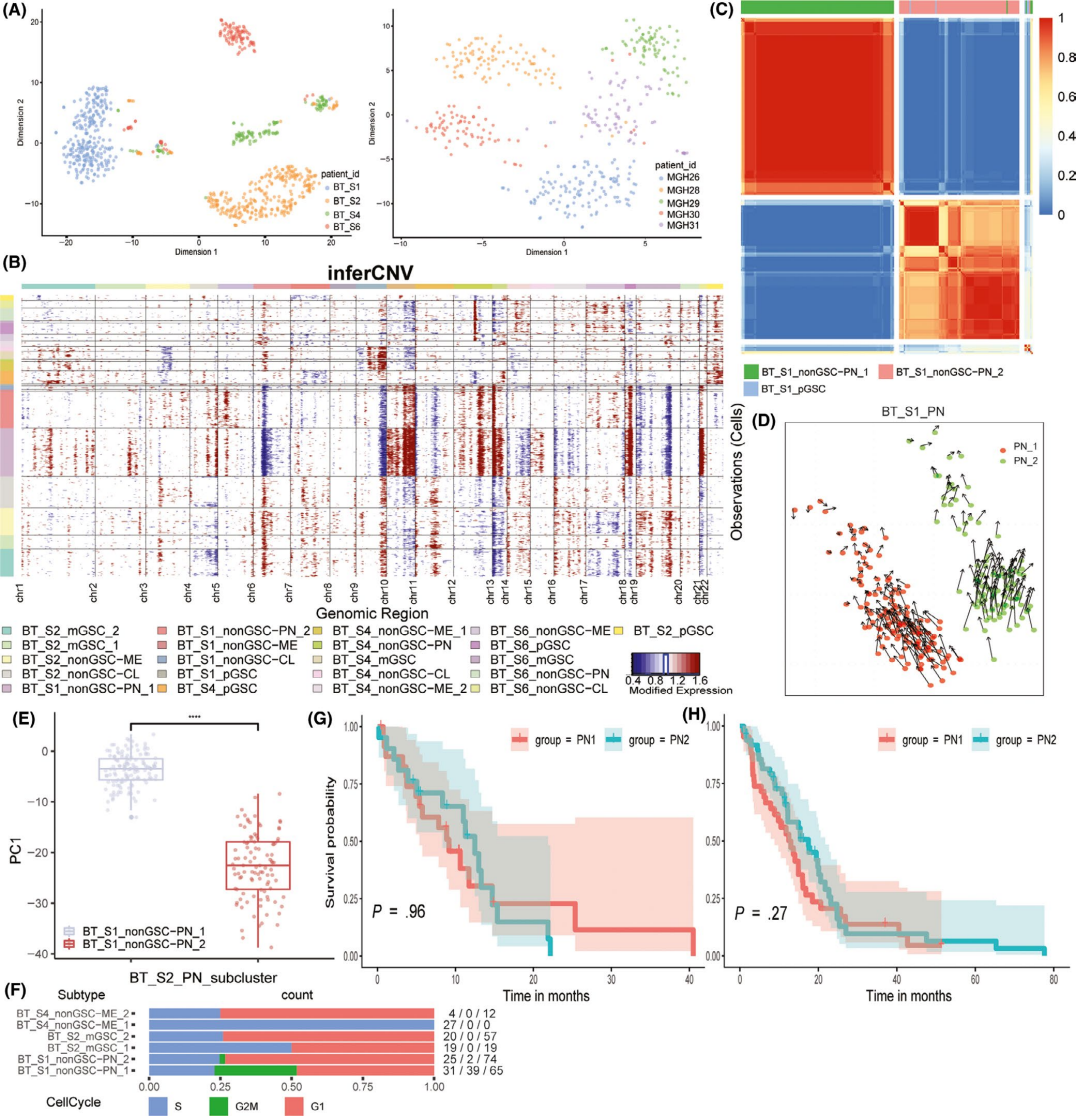
A, the tsne plot of neoplastic cells annotated by patient ID. B, inferred CNV based on neoplastic cells scRNA‐seq divided by patient ID and subtypes. Red means amplification and blue indicates deletion. C, consensus cluster within patient BT_S1 PN subtype of cells, subclones were clustered together. D, transition direction predicted by RNA velocity in patient BT_S1 PN subclones. E, PC1 values’ distribution of patient BT_S1 PN subtype of cells. F, proportion of cells in different cell cycle stages of neoplastic cells in each patient G, Kaplan‐Meier curve of primary IDH wild‐type glioblastoma in TCGA database (Hiseq). H, Kaplan‐Meier curve of primary IDH wild‐type glioblastoma in TCGA database (microarray)

R packages survival and survminer were used for overall survival analysis. All statistical analyses were performed using R software, version 3.6.2 (The R Foundation for Statistical Computing, http://www.rproject.org/). The Shapiro‐Wilk method was used for normality test. The p‐values for the significance of comparison among groups in Figure 1F, 2E, 4A were calculated using the Wilcoxon rank‐sum test. The p‐value for the significance of PDCD1LG2 between groups was calculated using Student's *t* test.

## 3 RESULTS

### 3.1 Intra‐tumoral heterogeneity and cell subtype identification

We removed 473 cells in the GSE84465 dataset whose total counts were <25 000 or total gene number were >6000 to reduce the bias caused by low‐quality sequencing or double‐cell contamination (Figure S1A‐B). Thereafter, we selected neoplastic cells for further analysis. Compared to the four subtypes proposed by Verhaak, three subtypes classification improved by Wang based on Verhaak subtypes could obviously decrease the MIX cell number (Figure 1A). Consistent results were also achieved in the validation (GSE57872) dataset (Figure S1C). We then identified mGSCs and pGSCs in GSE84465. mGSCs were derived from ME and CL subtypes, while pGSCs were mainly derived from PN subtype (Figure 1B). Additionally, the MIX subtype in Verhaak subtype was mainly identified as GSCs (Figure 1C). Finally, five subtypes were obtained: mGSCs, CL, ME, PN, and pGSCs in the dataset. After PCA analysis of all neoplastic cells, genes that contributed to PC1 (genes with top 20% PC1 values) were enriched at mitosis‐related pathway (Figure 1D). mGSCs were weaker than pGSCs, and PN was the most active subtype in mitosis (Figure 1F and S1D). In terms of differential potency, mGSCs possessed the highest potency and PN was the lowest followed by pGSCs (Figure 1E). RNA velocity showed that GSC subtype transitions from mesenchymal to proneural phenotype (Figure 1G).

### 3.2 Inter‐tumoral heterogeneity dominated heterogeneity of primary IDH wild‐type glioblastoma

All neoplastic cells were pooled by the patient ID instead of identified subtypes, and validated in the validation dataset (Figure 1A,C and Figure 2A). However, within patients, cells had clear distinction among different subtypes (Figure 1A,C). We used the hidden Markov model to infer the CNV status through single‐cell RNA‐seq data. The results showed that the CNV phenotypes were more different between patients, and different subtypes within the same patient shared more commonality than between patients (Figure 2B and S2A).

### 3.3 Heterogeneity in neoplastic subtype of cells of the same patient

Even in the same patient, the same subtype of cells had different subclones, such as patient BT_S1 PN_1 and PN_2 cells (Figure S2B), patient BT_S2 mGSC_1 and mGSC_2 cells, and patient BT_S4 ME_1 and ME_2 cells. Moreover, within the same patient and same subtype, these subclones clustered together (Figure 2C and S2C,D). To infer the subclones’ state transformation relationship within subtypes, we used RNA velocity algorithm and found that within patient BT_S1, PN_1, and PN_2 seemed to be different differentiated cells from the same origin (Figure 2D), while BT_S2 mGSC_1 and mGSC_2 and BT_S4 ME_1 and ME_2 had state transition relationship with each other (Figure S2E,F). To examine differences between PN subclones, we used PCA on BT_S1 PN cells, and PC1 could distinguish between PN_1 and PN_2 with good performance (Figure 2E). We chose the top and bottom 20% genes based on PC1 values to conduct GO enrichment, which showed that PN_1 did well in development growth while PN_2 did well in the catabolic process (Figure S2G,H). The cell cycle prediction supported the differences between PN subclones, which also showed that subclones within patient‐subtypes had divisional differences (Figure 2F). We also explored whether the different subclone percentages impact prognosis, by using top and bottom 100 genes ordered by PC1 values of PN cells as PN_1 and PN_2 markers, which were used to calculate PN_1 and PN_2 ssGSEA scores in TCGA Hiseq and microarray dataset of primary IDH‐wild type PN subtype GBM samples. We calculated the subtraction of PN_1 and PN_2 ssGSEA scores and defined the sample with score ≤0 as PN_1‐like tumor and the other as PN_2‐like tumor. No survival difference was observed between the groups (Figure 2G,H).

### 3.4 Regulon difference was mainly affected by inter‐tumoral heterogeneity

**FIGURE 3:**
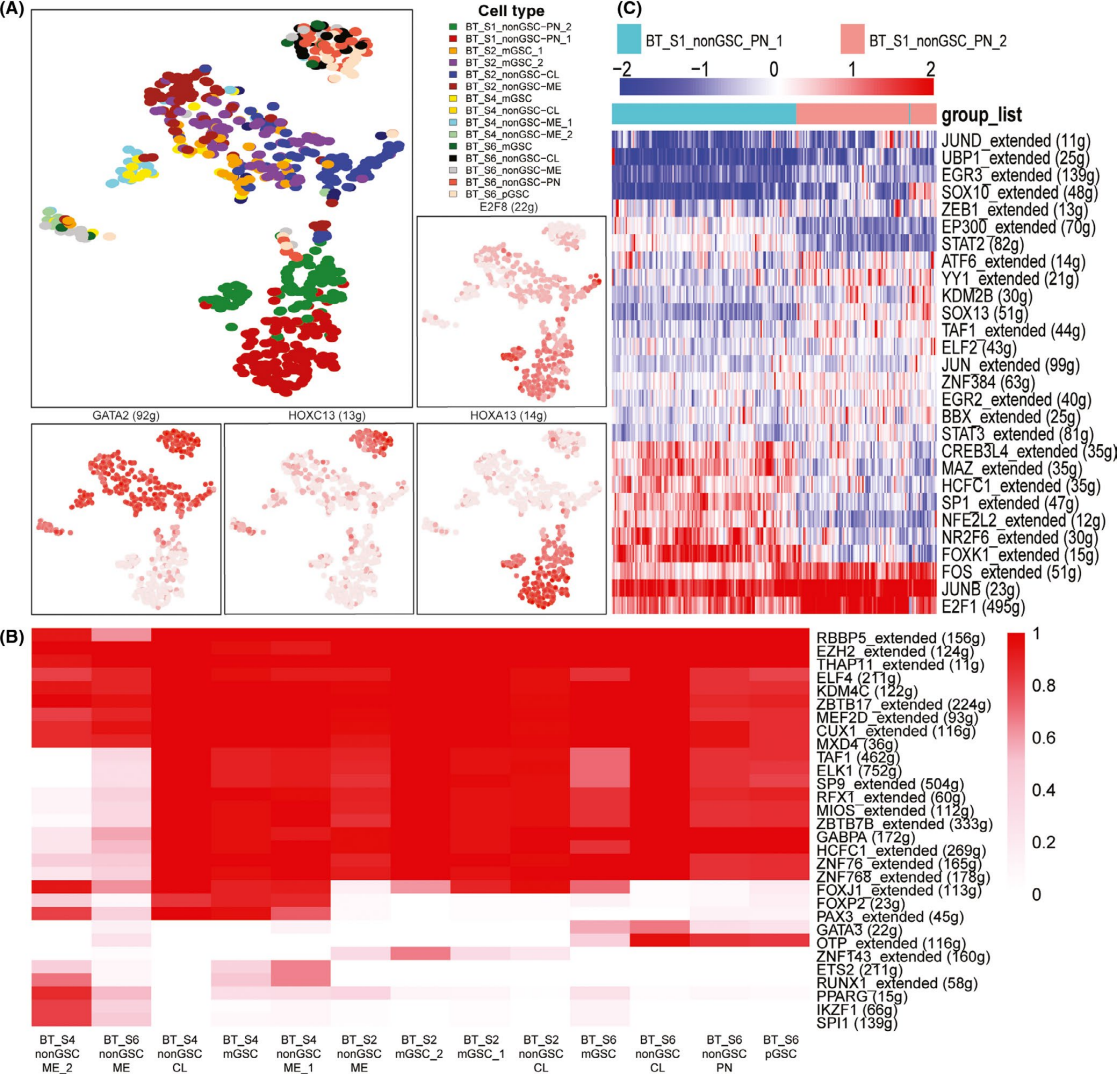
A, neoplastic cells of all patients clustered by regulon activity. The red of the surrounding plots represents the activity of regulons (AUC). B, heat map of binarized regulon network activity, red means regulon on and blank means regulon off. C, patient BT_S1 PN subclone cells hierarchy clustered by regulon activity (AUC). The heat map list only the regulons with significant differences

To compare the transcription factor regulon differences among patients, subtypes, and subclones, we adopted SCENIC to calculate the regulon network. Based on the regulon network, the main difference in regulon existed among patients (Figure 3A,B and S3A). Despite the mixing of GSCs and non‐GSCs within the same patient, the regulon network of different non‐GSCs subtypes (CL, ME, PN) and subclones were divergent. Since BT_S1 PN cells shared little regulon with other patients and cells, we selected these cells and found that the regulon activity could also distinguish subclones of the same subtypes within patients (Figure 3C). The subtypes and subclones of other patients performed the same as mentioned for patient BT_S1. Additionally, we observed that regulon networks could indicate the cell lineage trajectory of subtypes and subclones within the same patient. Even though subclones were identified as the same subtype, they possessed different transition directions. In regulon network of patient BT_S2, mGSC_1 was closer to CL than ME cells and mGSC_2 was closer to ME cells (Figure 3B). The lineage trajectory of this patient's neoplastic cells showed that some mGSC_2 cells differentiated to mGSC_1 cells, the progenitor of CL cells in this patient, and the remaining mGSC_2 cells differentiated to ME cells (Figures S2E and S3B). Within patient BT_S4, the regulon network of mGSCs was closer to ME_1 than CL. Its lineage trajectory supported the regulon network differences among subtypes and subclones (Figure S3C). Patient BT_S4 ME_2 cells and patient BT_S6 ME cells shared more regulons, which could not be separated by tsne (Figure S3D).

### 3.5 Subtype‐specific cell‐cell communication was rarely affected by inter‐tumoral heterogeneity

**FIGURE 4:**
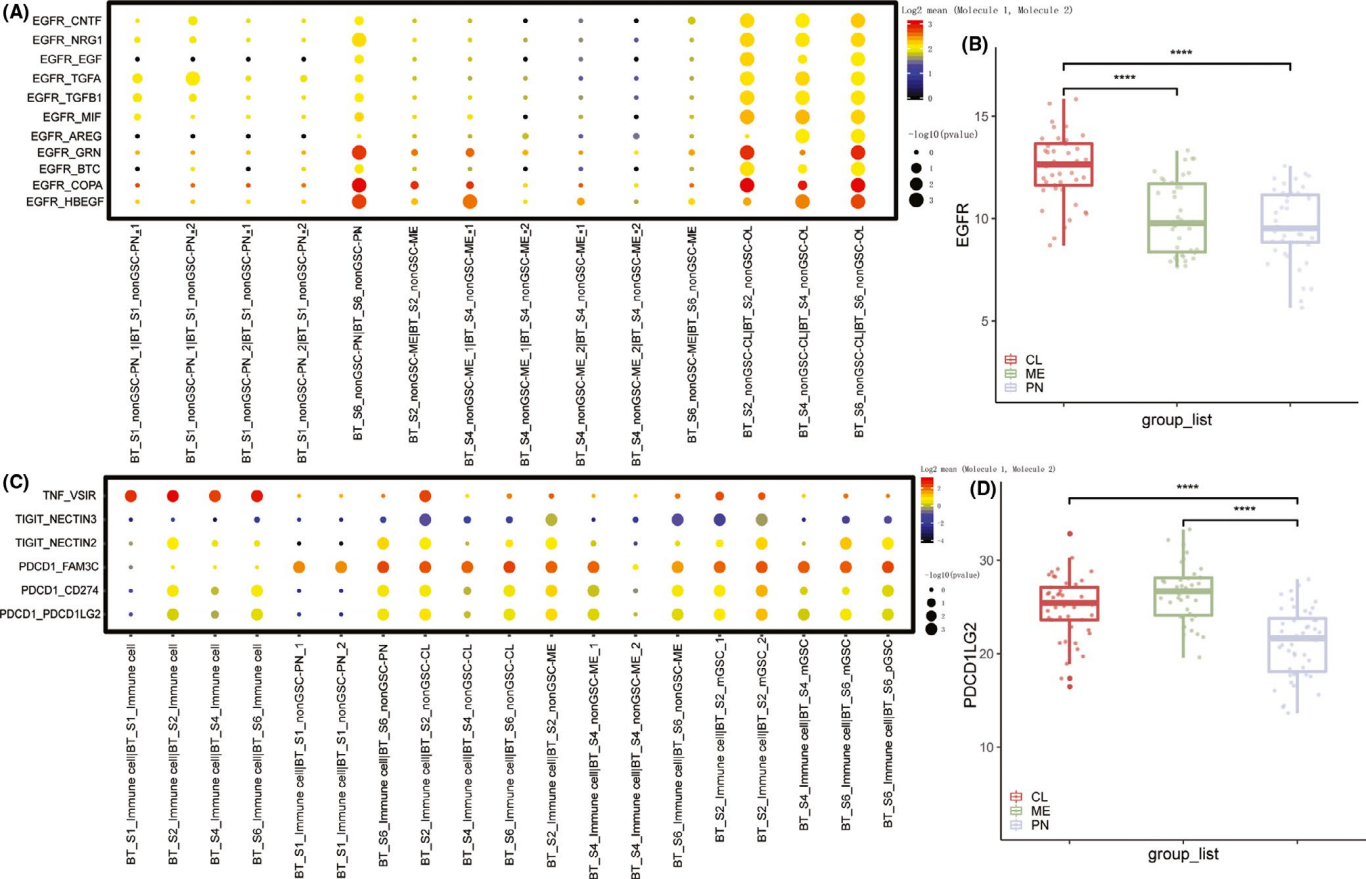
A, the receptor‐ligand interaction (EGFR) within each subtype of each patient. B, EGFR expression of each subtype primary IDH wild‐type glioblastoma from TCGA database. C, the receptor‐ligand interactions between immune cells of each patient and neoplastic subtype of cells. D, the expression of PDCD1LG2 of each subtype primary IDH wild‐type glioblastoma from TCGA database

To examine the cell‐cell communication difference among each subtype within patients and identify commonality within each subtype among patients, we used CellPhoneDB to infer the unbiased receptor‐ligand interaction among cells. Within all patients, CL cells expressed a high level of ligands of epidermal growth factor receptor (EGFR) interacting with EGFR receptors on CL cells (Figure 4A), which was consistent with CL subtype characteristics in bulk samples^10^ (Figure 4B). Interaction within CADM1, involved in neuronal migration, axon growth, pathfinding, and fasciculation on the axons of differentiating neurons, was strong in PN subtypes and mesenchymal cell mitogens PDGFB, ME subtype marker MET, and TNF, TNFRSF1A, and TNFRSF1B involved in tumor necrosis factor superfamily pathway expressed specifically in ME subtype were highly interactive within ME cells^10^ (Figure S4A,B). In addition, we also evaluated immunosuppressive interactions between subtypes and immune cells in each patient. Intra‐immune‐cell interactions would activate the immunosuppressive receptors, like VSIR, among all patients. In terms of immunosuppression between subtypes and surrounding immune cells, PN cells had weaker effects than ME and CL cells, and PDCD1LG1 expression was consistent, the ligand of PDCD1, from the TCGA dataset (Figure 4C,D).

## 4 DISCUSSION

Intra‐tumoral heterogeneity that involves different subtypes of cells within a tumor provides different insights into tumor biology. Previous research has confirmed the intra‐tumoral heterogeneity in primary IDH wild‐type glioblastoma by single‐cell analysis.^8^ In bulk samples, GBM could be divided into four subtypes: CL, ME, PN, and NE. However, recent research found that the NE subtype is nontumor specific, which is caused by normal NE tissue contamination surrounding the tumor margin and tested these subtype classification also suit GBM neoplastic cells.^10^, ^15^, ^23^ Meanwhile, there are very few strong markers of glioma stem‐like cells (GSCs) due to the limitation of bulk samples. Single‐cell sequencing offers a feasible way to identify the GSCs and reveal the lineage relationships among GSCs and non‐GSCs. Lin et al revealed that GSCs mainly contain two subtypes: mesenchymal GSCs (mGSCs) and proneural GSCs (pGSCs) because CL samples can be distinguished from ME samples due to different cell infiltrations.^16^ We applied their GSC markers and identified pGSCs and mGSCs in patients, and the characteristics of different GSCs were analyzed, such as GSC division activity and transition direction between GSCs. The results of this study were consistent with previous results, which proves the validity of this identification. Since Verhaak subtypes of GBM were discovered based on bulk samples, we thought it might be the reason for most GSCs being identified as the MIX group when Verhaak subtypes were identified in single‐cell data. The renewed three subtypes’ classification discovered based on both bulk and single‐cell data was more suitable for GBM non‐GSC neoplastic cell classification.

Despite the intra‐tumoral heterogeneity in GBM, we found that tumor heterogeneity was mainly due to inter‐tumoral heterogeneity. Although neoplastic cells were classified as the same subtype, these cells from the same patient clustered together and their CNV status, as well as regulon network, shared more similarity within the same patient. Our results indicated that not only the DNA structural variation but also the transcription factor regulon difference of GBM were mainly influenced by the genetic background of patients. The study on GLASS cohort revealed few common features of glioma evolution across subtypes and instead showed highly variable and patient‐specific trajectories of genomic alterations.^24^ According to the COSMIC signature database,^25^ the dominant mutational signature in IDH‐wild type glioma was aging, which indicated that aging majorly contributed to the differences of regulon and genetic alterations among IDH wild‐type glioblastoma patients.^24^

Within the same patient, though different subtypes of cells shared many commonalities in CNV changes and regulon network, there were differences to some degree. We found that the non‐GSCs, which shared more common features in CNV or regulon network with a specific GSC, were derived from this GSC by trajectory analysis, suggesting that the differentiation or evolution of tumor cells is a gradual process with mutation accumulation.^26^ We also identified the subclones of the same subtype in the same patient. In patient BT_S1, PN subclones shared few commonalities in the GO pathway and were two different subclones from the same progenitor. The subclones belong to distinct cell lineages could coexist in the same malignancy due to tumor evolution, and the distribution of this patient's CNV indicated that the GBM was in the inferred mid growth phase.^27^ We did not find negative survival impacts of PN_1 and PN_2 on primary IDH wild‐type glioblastoma patients in TCGA and hypothesize that the subclones emerged due to neutral evolution, which dominated in cancer evolution, and both had not acquired the necessary alterations for progression.^28^, ^29^ mGSC subclones existed in patient BT_S2, and the mGSC_1 subclone was derived from mGSC_2 subclone. Regulon network similarity among mGSC_1, mGSC_2, CL, and ME cells was consistent with their lineage trajectories, which indicated that some mGSC_2 differentiate to mGSC_1 and then transition to CL cells, and the remaining mGSC_2 transition to ME cells. This finding was consistent with previous research that ME and CL cells are derived from mGSCs.^16^ We also discovered that BT_S4 ME subclones ME_2 shared more similarity with BT_S6 ME cells, which could not be distinguished by tsne, instead of BT_S4 ME_1 subclone. It indicated that some subclones were less affected by inter‐tumoral heterogeneity and we speculated that these subclones may be directly derived from driver mutations.^30^, ^31^ In patient BT_S4, ME_2 showed a tendency of transition to ME_1 subclones, and we thought that these two subclones possessed the same progenitor and were at different phases of glioma genesis of one consecutive evolution process.^26^

Since the accumulation of alterations in GBM cells occur over decades‐long growth phase that leads to a highly diverse population,^32^ we thought each subtype of cells within patients that clustered together and showed similar CNV status were from the same clonal expansion and at the same anatomical region.^1^, ^23^ Thus, we used CellPhoneDB to infer the inner cell communication of each subtype by receptor‐ligand interaction within the same subtypes. CL cells highly expressed EGFR ligands interacting with EGFR on themselves, which was consistent with the results that EGFR was frequently amplified in the CL subtype.^10^, ^23^ CADM1 played a pivotal role in developing neurons and highly interacted within PN subtypes, indicating the relationship between neural stem cells and PN progenitor.^33^, ^34^ Interactions within ME cells mainly occurred in tumor necrosis factor superfamily pathway, which promoted ME cell differentiation and radio resistance.^35^ When inferring the immunosuppressive interaction with the immunosuppressive receptor, including PDCD1, TIGIT,^36^ and VSIR,^37^ between neoplastic subtypes of cells and immune cells, the immunosuppressive effect of ME was higher than other subtypes of cells, which suggested that ME had higher TAM infiltration than other subtypes.^38^

In summary, compared to intra‐tumoral heterogeneity, inter‐tumoral heterogeneity contributes more to tumor heterogeneity of primary IDH wild‐type glioblastoma. The subtype molecular characteristics were based on the patient's genetic background, and different subtypes of cells share more molecular similarities within the same patient than the same subtype of cells in different patients. However, although neutral mutation accumulation in patients contributes to different genetic backgrounds, the driver mutations and cell‐cell communication of subtypes of cells remained stable among patients.^24^, ^28^

## CONFLICT OF INTEREST

The authors declare no conflict of interest.

## Supporting information

Figure S1

Figure S2

Figure S3

Figure S4

Supplementary Material
