# Supplementary figures and images for: Effect of intra‐ and inter‐tumoral heterogeneity on molecular characteristics of primary IDH‐wild type glioblastoma revealed by single‐cell analysis
Source: CNS Neurosci Ther. 2020 Jun 2;26(9):981–9. doi: 10.1111/cns.13396 (PMC7415209; doi:10.1111/cns.13396)

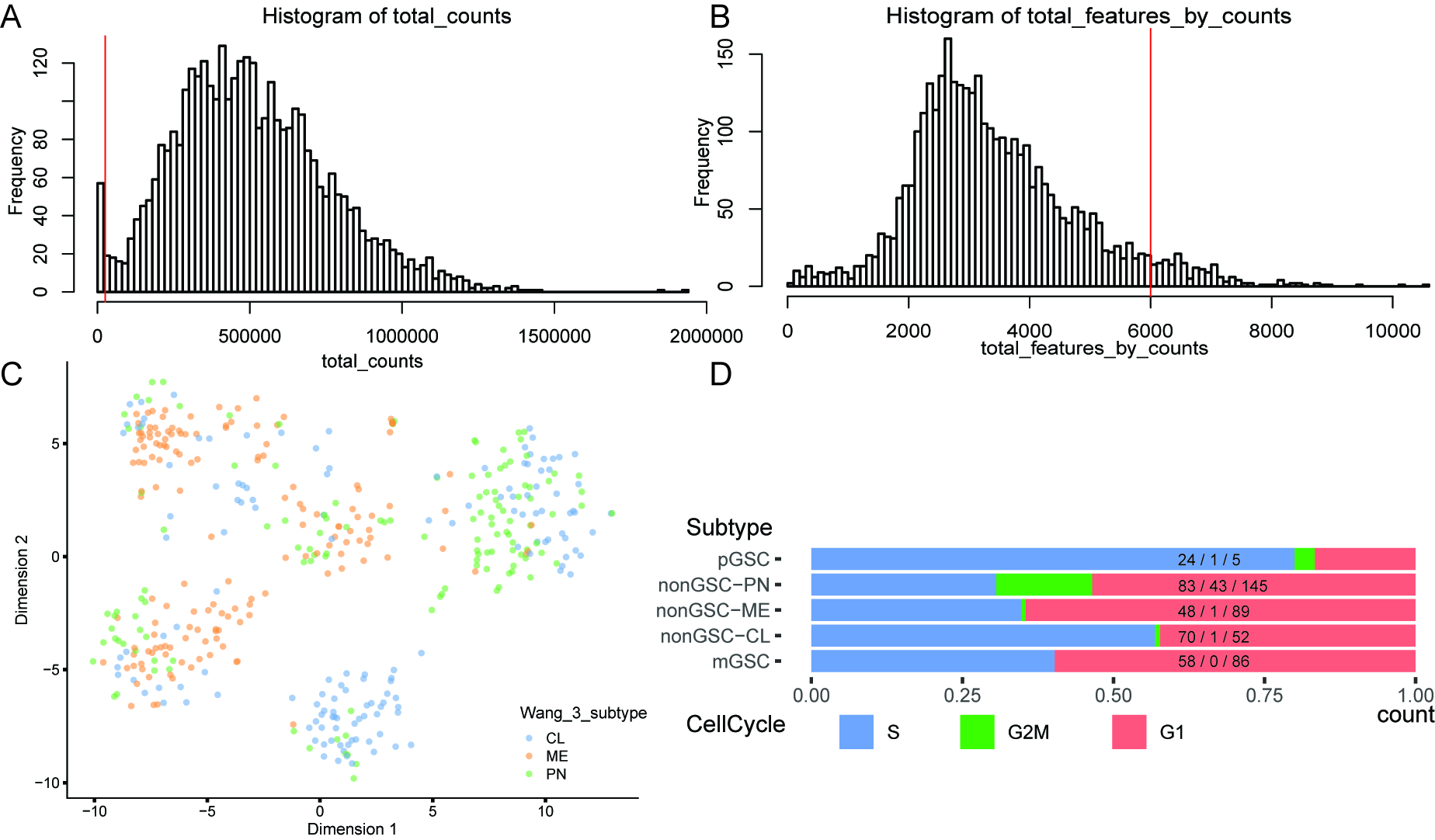

Supplement: Supplementary file 1 — Figure S1 [file CNS-26-981-s001.tif]

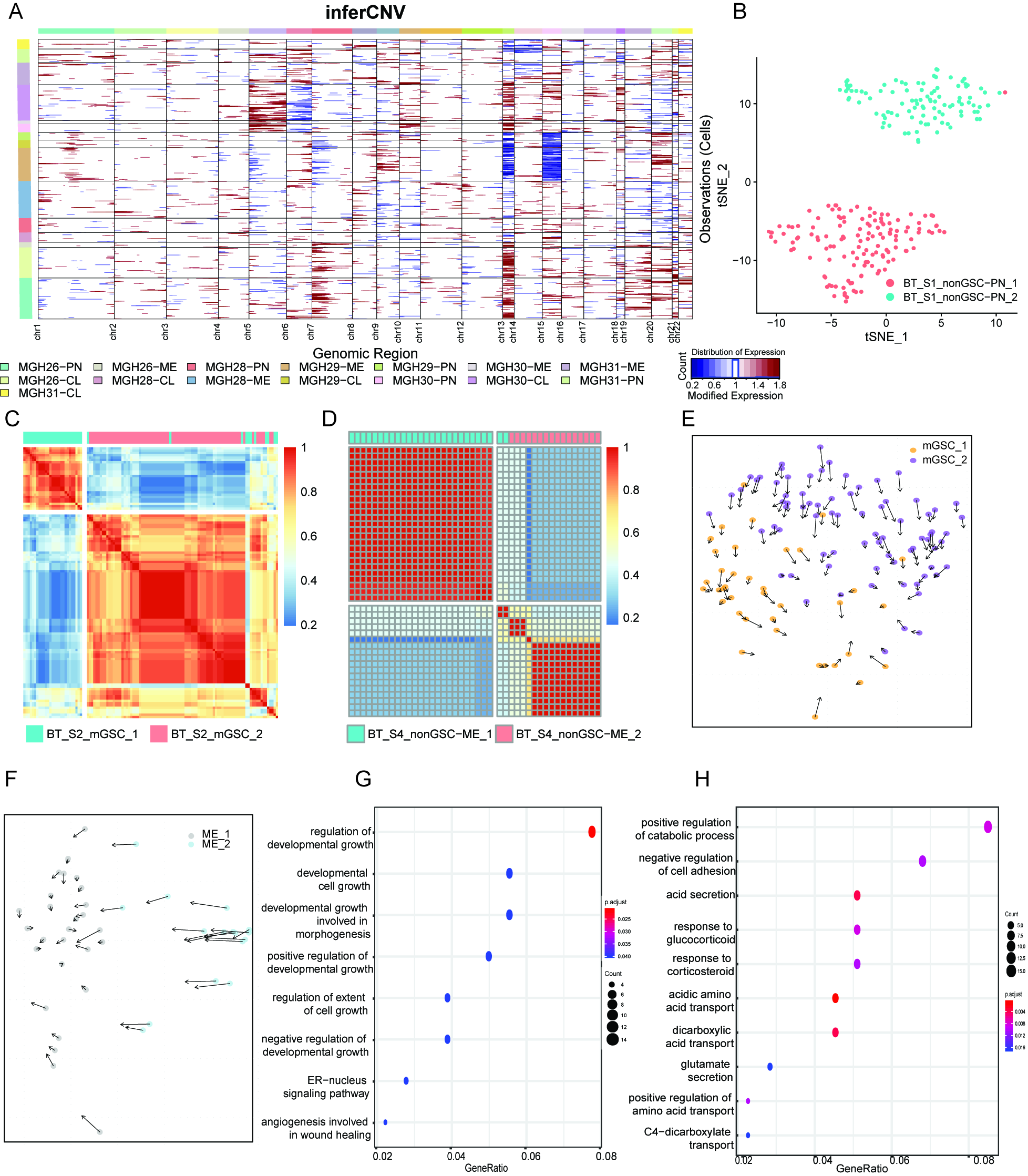

Supplement: Supplementary file 2 — Figure S2 [file CNS-26-981-s002.tif]

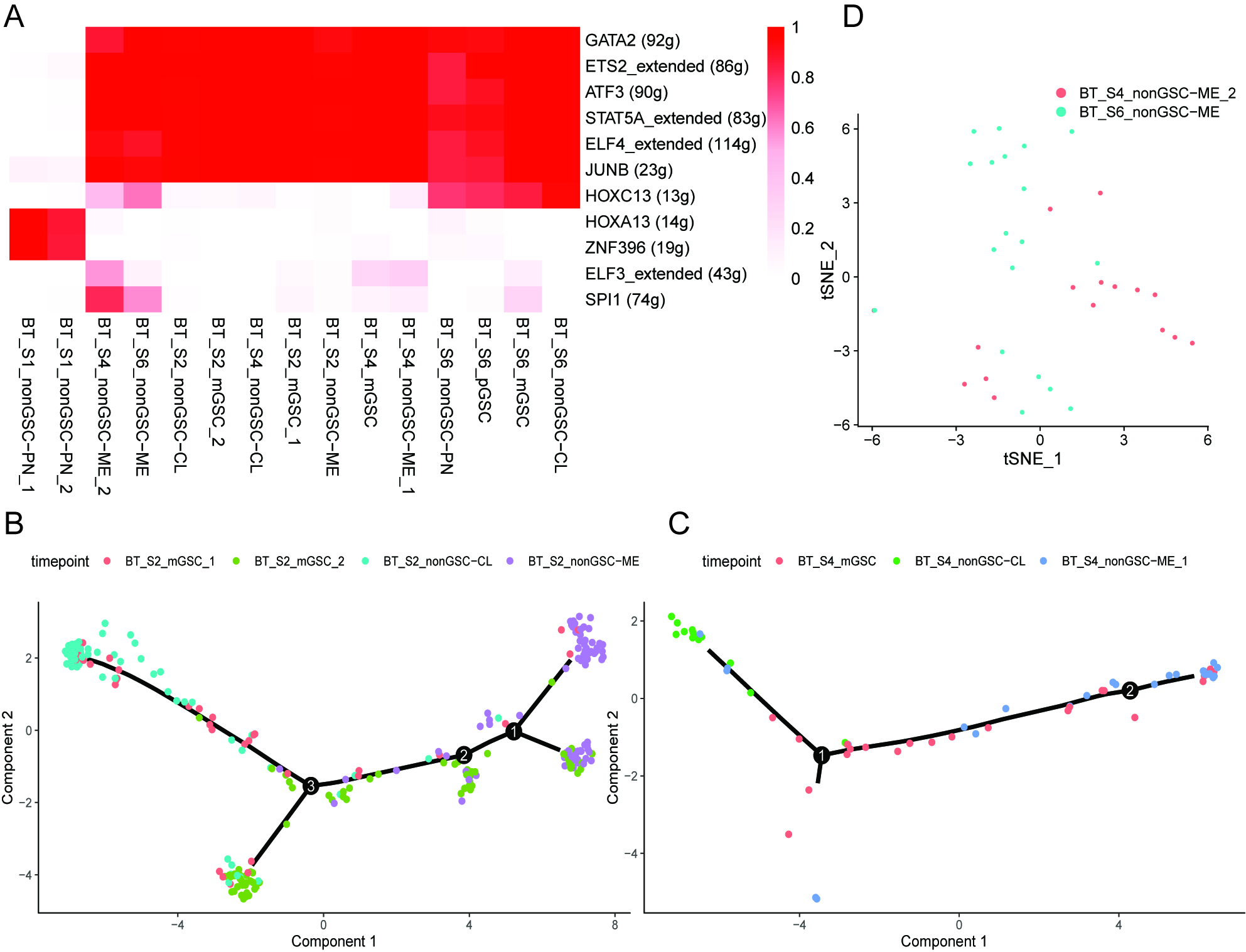

Supplement: Supplementary file 3 — Figure S3 [file CNS-26-981-s003.tif]

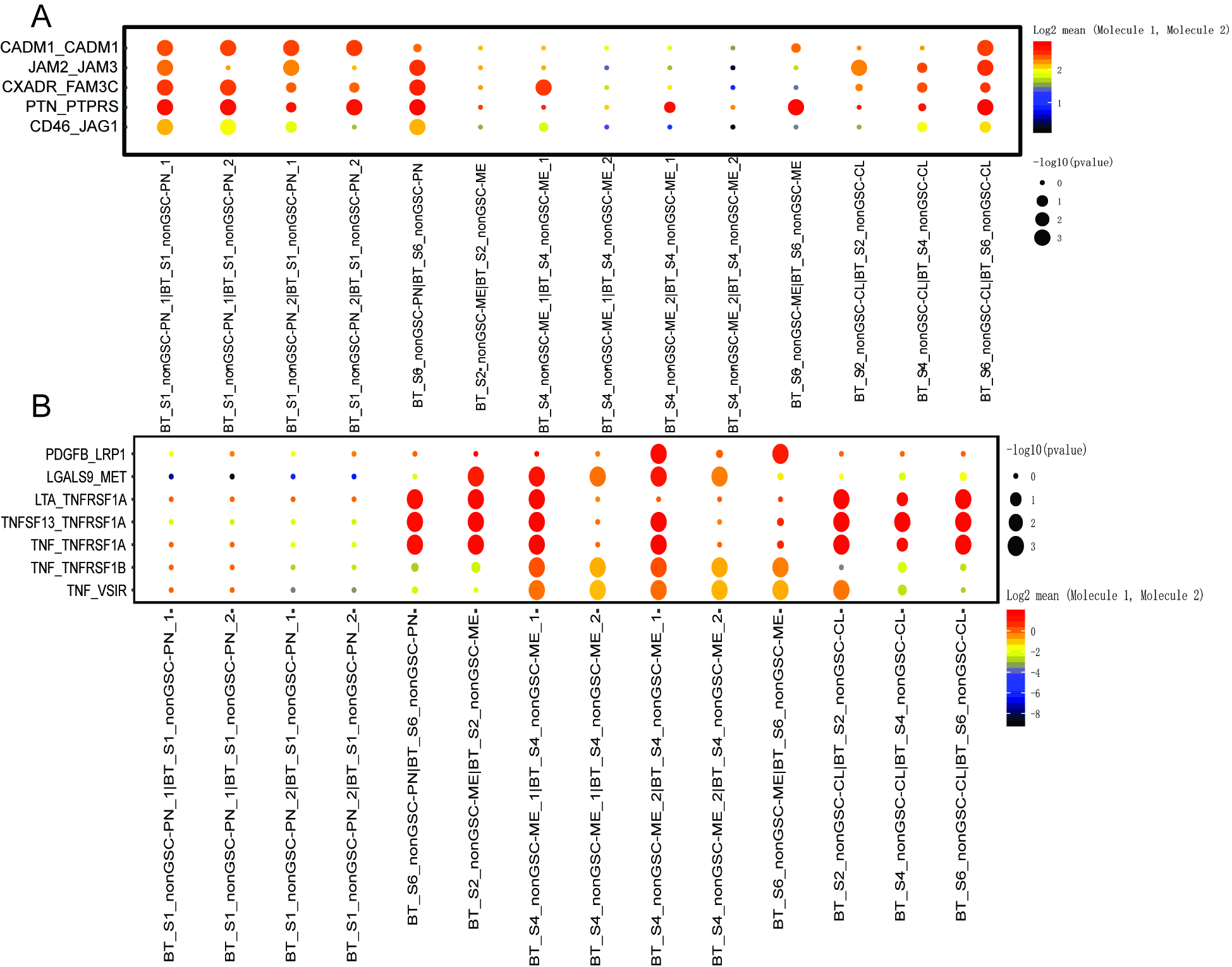

Supplement: Supplementary file 4 — Figure S4 [file CNS-26-981-s004.tif]
