# Supplementary material for: Effect of intra‐ and inter‐tumoral heterogeneity on molecular characteristics of primary IDH‐wild type glioblastoma revealed by single‐cell analysis
Source: CNS Neurosci Ther. 2020 Jun 2;26(9):981–9. doi: 10.1111/cns.13396 (PMC7415209; doi:10.1111/cns.13396)
Supplement: Supplementary file 5 — Supplementary Material [file CNS-26-981-s005.docx]

Figure S1. A, the distribution of total counts of scRNA-seq data, the cells with counts <25000 were filtered out. B, the distribution of total genes of scRNA-seq data, the cells with genes >6000 were filtered out. C, neoplastic subtypes identified in neoplastic cells from GSE57872 dataset. D, proportions of cell cycle states of each neoplastic subtype.

Figure S2. A, inferred CNV grouped by patient-subtype in GSE57872. B, the tsne plot of patient BT_S1 PN subclones. C, consensus cluster of patient BT_S2 mGSC subclones. D, consensus cluster of patient BT_S4 ME subclones. E, transition direction inferred by RNA velocity between patient BT_S2 mGSC subclones. F, transition direction inferred by RNA velocity between patient BT_S4 ME subclones. G, GO enrichment pathway of 200 genes with top PC1 values. H, GO enrichment pathway of 200 genes with bottom PC1 values.

Figure S3. A, heat map of binarized regulon activity (AUC) in all patients and neoplastic subtypes of cells. B, lineage trajectory among mGSCs, ME cells and CL cells within patient BT_S2. C, lineage trajectory among mGSC, ME cells and CL cells within patient BT_S4. D, the tsne plot of patient BT_S4 ME_2 cells and patient BT_S6 ME cells.

Figure S4. A, the receptor-ligand interaction, which was active in PN cells, within each subtype of each patient. B, the receptor-ligand interaction, which was active in ME cells, within each subtype of each patient.
